# Removal of methylene blue from synthetic industrial wastewater by using geopolymer prepared from partially dealuminated metakaolin

**DOI:** 10.1038/s41598-025-01461-w

**Published:** 2025-05-21

**Authors:** Khaled Elewa, A. F. Tawfic, Mostafa Tarek, Nabil Abdullah Al-Sagheer, Nabil M. Nagy

**Affiliations:** 1 Department of Civil Engineering, Military Technical College, Cairo, Egypt; 2 Head of Nuclear Engineering Department, Military Technical College, Cairo, Egypt; 3 Aluminum Sulfate Co. of Egypt, Cairo, Egypt

**Keywords:** Adsorption, Geopolymer, Industrial wastewater treatment, Metakaolin, Methylene blue removal, Silicious waste of aluminum sulfate-based kaolin, Environmental sciences, Environmental chemistry, Environmental monitoring, Pollution remediation

## Abstract

Industrial wastewater frequently contains a huge of pollutants, such as heavy metals, dyes, and organic compounds, leading to considerable environmental discharge. Methylene Blue (MB), a cationic dye extensively utilized in the textile and pharmaceutical sectors, presents significant hazards to aquatic environments and human health. Exposure to MB may result in negative effects, including dermal irritation, stomach discomfort, and possible long-term ecological consequences. This research investigates the elimination of MB from synthetic industrial wastewater utilizing a geopolymer derived from Partially De-aluminated Metakaolin (PDK) by using adsorption technique it is a simple, low-cost, and effective method for removing a variety of pollutants. The geopolymeric materials were analysed using XRF, XRD, FTIR, SEM, EDX, and BET to confirm its structure then used for MB removal. The specific surface area of the geopolymer was determined to be 9.3 m^2^/g and a pore volume of 0.024 m^3^/g. Its kinetics and isotherms were investigated in the MB dye adsorption experiments. The adsorption was influenced by dose, initial dye concentration, pH, and contact time. According to the results, the maximum adsorption capacity (8 mg/g) was attained at 60 mg/L MB, 60 min of contact time, and pH 7–12 for one hour. The experimental data indicated that Freundlich isotherm model was the best-fit model to describe the sorption of MB on the synthesised geopolymer with higher determination coefficients R^2^ of 0.996. Value of n greater than unity indicates a favourable adsorption taking place. This indicated that the adsorption occurs under a multilayer and heterogeneous surface for MB. The adsorption kinetics of MB onto GP was investigated using pseudo first order, and pseudo second order models as shown in, using the experimental data at various initial concentrations. The calculated parameters values obtained from the application of three models are tabulated. By comparing R^2^ for each applied model and the compatibility between the estimated and observed q_e_ values, the most favourable model may be identified. The pseudo 2^nd^ order R^2^ value are greater than other models.

## Introduction

**Fig. 1:**
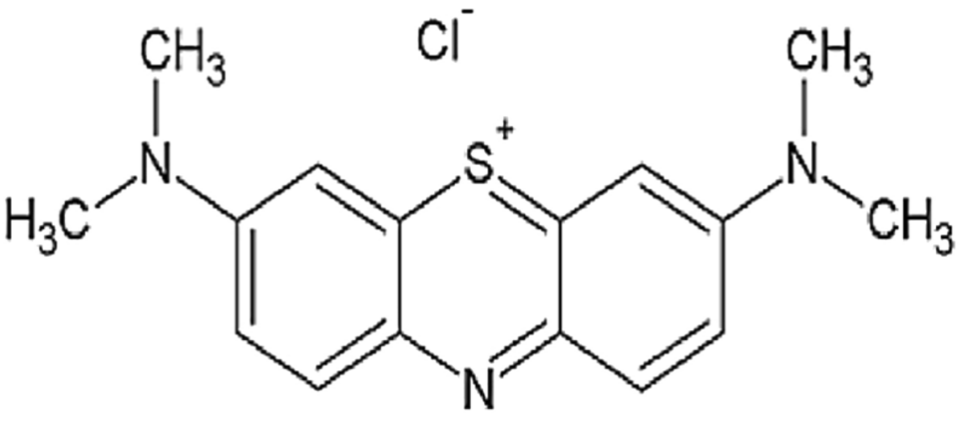
The molecular structure of MB^17^.

The treatment of effluents has become a difficult problem in environmental sciences as a result of the rapid industrial growth that has caused the discharge of many colours into the aquatic environment. The extensive use of dyes across various sectors, including textiles, leather, additives, petroleum products, paper, cotton, wool, plastics, and pharmaceuticals, has led to increased water pollution from colorants, garnering the attention of the scientific community.^1–4^. Dyes are utilized on substrates to give them a persistent coloration that withstands fading from exposure to water, light, oxidizing chemicals, perspiration, and microbiological assault^5–7^. Owing to these benefits, a variety of dyes are employed across numerous industries, including textiles, food, rubber, printing, cosmetics, pharmaceuticals, plastics, concrete, and paper, for diverse applications^8–11^. These companies produce significant volumes of wastewater filled with carcinogenic and poisonous dyes, rendering the water unsuitable for human consumption^12^. Methylene blue (MB), one of the most used industrial dyes, it has a molecular weight and chemical formula of 319.85 g/mol and C_16_H_18_N_3_Sl (Fig. 1), is a basic dye that is utilized in many different industries, including color photography, textile dyeing, and the petroleum sector. For this reason, treating water that has been tainted by toxic chemicals is essential for both environmental preservation and the repurposing of these unusual water^13–16^.

Various physical processes exist for the removal of dyes from wastewater discharges. Among the most effectively employed methods are filtration processes (membrane, nanofiltration, ultra/microfiltration), reverse osmosis, ion exchange, irradiation, electrolysis, coagulation-flocculation, and adsorption techniques^18–21^. Solid sorbents were utilized in adsorption technique to remove dyes like MB. The method has been used extensively and successfully to extract MB from wastewater. Numerous adsorbents have been studied and successfully used to lower the concentrations of dyes in aqueous solutions^22^. This organic material was removed from aquatic media using a variety of natural and synthetic adsorbents, including magnetic chitosan, zeolite, kaolin^23–25^, activated carbon, natural clay, Fe_3_O_4_/activated montmorillonite nanocomposite, and kaolin^26–30^.

Among the synthetic adsorbents, geo-polymers are mostly utilized to eliminate organic materials^31^. In the 1970s, Professor Joseph Davidovits introduced the word “geopolymer” to the globe. The prefix “geo” stands for inorganic aluminosilicate, which is derived from geological sources and forms geopolymer through a polycondensation reaction with an alkaline solution^32^. The overall configuration of the geopolymer materials is mostly determined by the Si/Al ratio in the structural chains of the polymeric building blocks, which determines the three major forms of geopolymers^33,34^.

The selection of raw materials for the production of geopolymer is influenced by several aspects, including affordability, accessibility, application specificity, and end users’ express interest. Every source material has benefits and drawbacks. Poly-silicate include fine silica powder made from ferro-silicon metallurgy or sodium or potassium silicate supplied by the synthetic industry. to some extent Partially Dealuminated metakaolin (PDK) is a residual material produced after the chemical leaching of MK (such as calcined kaolin or Al_2_O_3_.2SiO_2_) in the aluminum sulfate industry^35^. The SiO_2_/Al_2_O_3_ ratio increased as a consequence of the dealumination procedure. The enhanced specific surface resulting from acid attack is attributed to the expansion of the pore volume, without a significant increase in the average pore size distribution^36^.

Clay is frequently used as the first ingredient to create geopolymers since it is inexpensive and readily available in large quantities^37–39^. Using kaolinite that has undergone pre-treatment and the resultant calcined metakaolin as the starting material for geopolymer synthesis is the subject of some noteworthy research on clay-based geopolymer^40,41^.

An essential role in the creation of geopolymer is played by the alkaline activator. The metal cation balances the negative charge in the framework formed by the tetrahedral aluminum, and the OH − ion can function as a catalyst in the process^42^. Because the Na^+^ ion is smaller and has a larger charge density than the K^+^ ion, it migrates across the gel network more quickly and efficiently, giving NaOH a stronger dissolving capacity than KOH^43^.

The analysis of PDK verified the existence of quartz and highly amorphous silica, which has a large surface area thereby suggesting a high level of reactivity. A typical consequence of acid attack on metakaolin is the breakdown of aluminum structures from octahedral and tetrahedral positions, resulting in the formation of unbound silica. Consequently, aluminum ions are being extracted from the lattice without disrupting its structure and exposing vacancies for replacement by other metal ions.

The purpose of this study is to efficiently synthesize of geopolymer from partially dealuminated metakaolin (PDK) which is an industrial residue of kaolin-based aluminum sulfate manufacturing process, and to present the using of the synthesized geopolymer for removal of methylene blue from wastewater. In this work the synthesized materials were characterized using several techniques to analysis the structure and morphology of the obtained geopolymer.

**Fig. 2:**
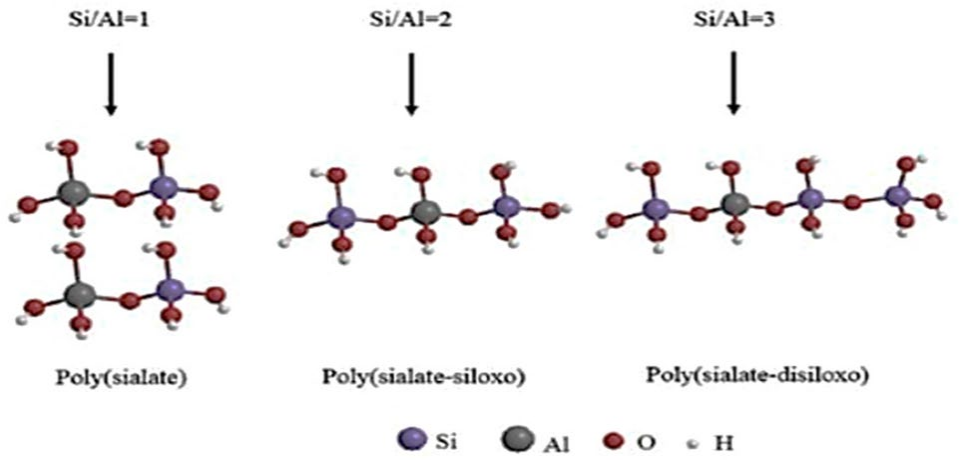
Patterns of aluminosilicate chains^44^.

This work synthesized the metakaolin-based geopolymer using a geopolymerization process. The developed adsorbent’s morphological and structural characteristics were described using FTIR, SEM, XRF, XRD, and BET analyses. Adsorbent dose, pH, contact time, and initial dye concentration, were among the several experimental settings used to examine the developed sample’s adsorption characteristics. In order to examine the batch adsorption process of the basic dye utilizing the synthesized metakaolin-based geopolymer, adsorption kinetics and isotherm data were examined (Fig. 2).

## Materials and experimental methods

### Materials

**Table:** 

Chemical	Source
Partial Dealuminated Metakaolin (PDK)	Aluminum sulfate factory, Egypt
Metakaolin (MK)	Aluminum sulfate factory, Egypt
Hydrochloric Acid (HCl), Purity 99.99%	Merck, Germany
Sodium Hydroxide (NaOH) Purity 99.99%	Merck, Germany
Methylene Blue (MB) Purity 99.99%	Merck, Germany

#### Preparation of geopolymer

**Fig. 3:**
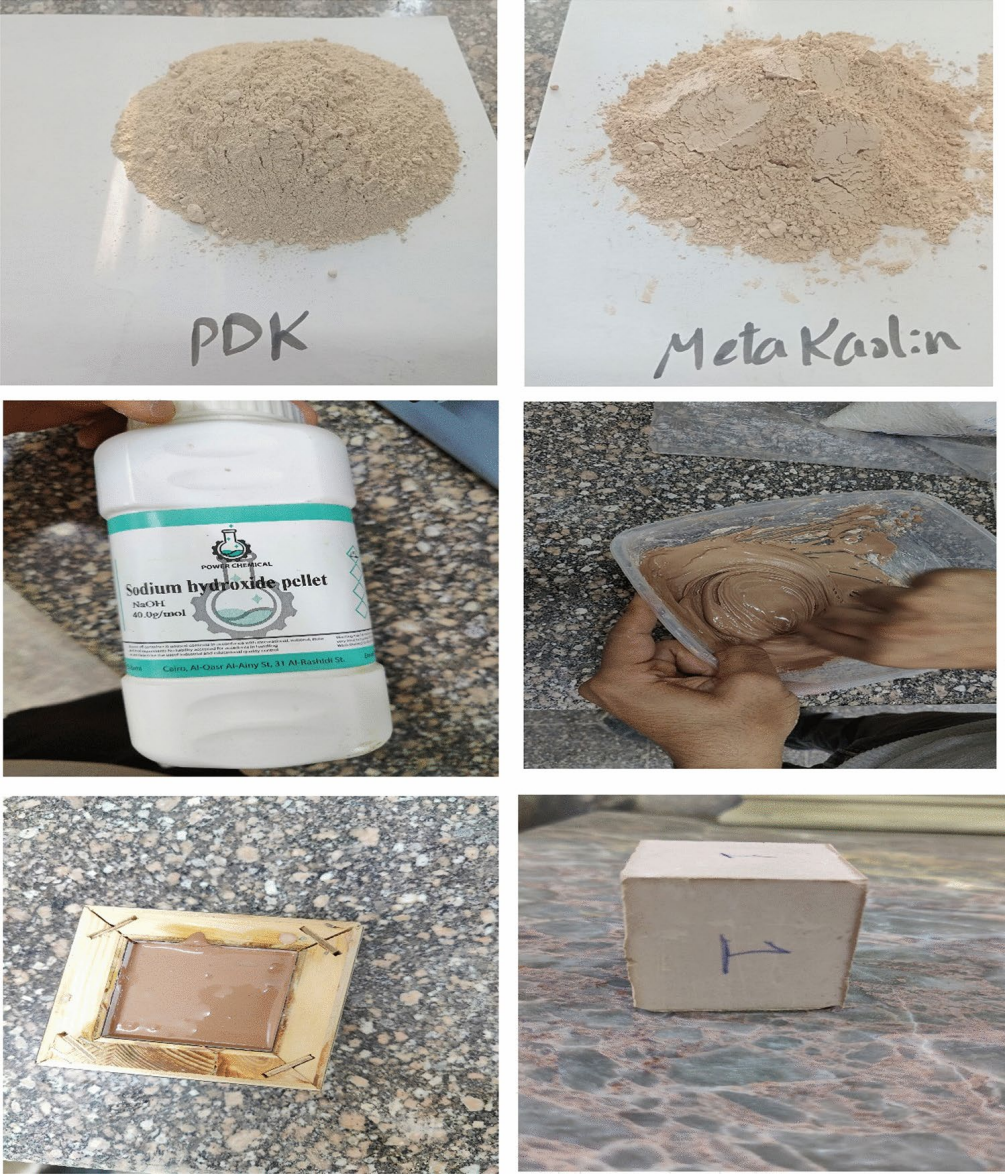
Preparation of geopolymer.

Figure 3 shows that the activator solution was prepared by adding of PDK powder to NaOH solution (12 M), this mixture was stirred at room temperature for period of 15 min. MK was added to the activator in a mixer at ambient temperature for 15 min with constant agitation to achieve optimal homogenization. The formed paste was mixed within ten minutes at room temperature before being poured into the cubic-plastic mold (50 × 50 × 50 mm) followed by drying at 70º C for 24 h. The formed geopolymer were ground and sieved to be between 63–200 μm, followed by washing in an excess amount of distilled water until the pH reached a neutral value. Compressive strength was measured to determine the mechanical properties of the geopolymer (Table 1).

**Table 1:** Geopolymer ingredients, and the mix design.

Sample	MK (g)	SiO_2_	Al_2_O_3_	Partially de-Aluminated metakaolin	SiO_2_	Al_2_O_3_	Total SiO_2_ Total Al_2_O_3_	NaOH (g)	Water (g)	Average Compressive Strength (MPa)	Molar ratio SiO_2_/Al_2_O_3_
				“Source of Silica” (g)							
#1	280	116.8 1.95 M	98 0.96 M	264	167.1 2.78 M	16.4 0.16 M	4.73 1.12	140	126	21	4.2
#2	330	137.6 2.29 M	115.5 1.13 M	205	129.8 2.16 M	12.7 0.125 M	4.45 1.26	165	110	28	3.5
#3	377	157.2 2.62 M	132 1.29	151	95.6 1.59 M	9.4 0.092	4.21 1.38	188	95	43	3.05
#4	425	177.2 2.95 M	148.8 1.46 M	94	59.5 0.99 M	5.8 0.057 M	3.94 1.52	212	80	32	2.6

## Characterization

Fourier Transform Infra-Red (FTIR) spectroscopy was applied to analyse the structure of geopolymers in combination with X-ray diffraction (XRD). This is because geopolymers are amorphous material, and cannot be described using only XRD. To confirm the formation of geopolymer and characterize the interaction between PDK in the presence of alkaline activation and MK, Fourier Transform Infra-Red (FTIR) spectra were recorded in a JASCO FT-IR/4100 spectrometer. The dried samples were ground with potassium bromide (KBr) in a portion of 1/10 (wt %) and scanned in the transmittance model. Spectra were taken from 4000 to 400 cm^−1^ wavenumber with a resolution of 2 cm^−1^.

The structure of PDK, and geopolymers such as the crystalline and amorphous forms was observed by XRD (Smart Lab, Rigaku, Japan) with Cu K_a_ radiation (*λ* = 1.54056 Å) at 40 kV and 30 mA. The XRD patterns were taken in the 2*θ* range from 10 to 40°, while the scanning speed was 0.01°/min. The surface topography and the structural composition of the samples were scanned using a Scanning Electron Microscope − Energy Dispersive X-Ray Spectroscopy. SEM (JSM-IT300, JEOL, Japan). Samples were coated with a thin layer of conducting material (gold) and imaged at various magnifications with an accelerating voltage of 10 − 15 kV. In addition, to observe the element distributions in the obtained geopolymers, an EDS (JSM-5300LV; JEOL Ltd., Japan) was also equipped.

M: Molar ratio.

Molar ratio = weight / molecular weight. For silica weight (g) divided by 60; For Alumina weight (g) divided by 102.

### Adsorption experiment of MB

A stock solution of MB (1000 mg/L) was prepared by dissolving MB in deionized water at a pH of 8. The adsorbent mass of the prepared geopolymer started with a dose of 350 mg and was placed in 50 mL of MB solutions containing 10, 20, 30, 40, 50, and 60 ppm. The mixtures were stirred for 180 min at 200 rpm and 298 K. After that, the sample was filtered with glass fiber filter paper. The initial and residual concentrations were measured using a UV−Vis spectrophotometer (V-750, Jasco, Japan) at a wavelength of 662 nm for MB. The removal percentage (R, %) of the geopolymers was determined using Eq. (1), and the adsorption capacity at any time *q*_t_ (mg/g) and adsorption capacity at equilibrium *q*_e_ (mg/g) were calculated using Eq. (2).

1 $${\text{R }}\left( \% \right) \, = {\text{ C}}_{o} \, - {\text{ C}}_{{\text{t}}} /{\text{C}}_{o} \times { 1}00$$

where R was the removal capacity of the dyes adsorbed (%), and C_o_ and C_t_ were the initial and equilibrium concentrations of dyes in the solution (mg/L), respectively.

2 $${\text{q}}_{{{\text{t}},{\text{ e}}}} = \, \left( {{\text{C}}_{{\text{o}}} \, - {\text{ C}}_{{\text{t}}} } \right) \, \times {\text{ V}}/{\text{M}}$$

where C_o_ and C_t_ (mg/L) were the concentrations of the solution at the initial time and a time t (h), respectively, C_e_ (mg/ L) was the equilibrium concentration of dye solution, V (L) was the volume of the contaminated solution, and M (g) was the weight of the dried adsorbent.

### Kinetic experiments

A Pyrex glass beaker with a volume of 2 L was used for kinetic process experiments. In the beaker, 1 L of MB aqueous solution at 50 mg /L was added and the pH was set to 7. The beaker cell’s centre was fixed with a three-bladed stainless-steel axial flow impeller. At room temperature, the impeller’s agitation was begun at 200 rpm. The exact weight of adsorbent was applied at zero-time interval. At various time periods, samples from the beaker were obtained. The ultimate equilibrium MB concentration of these samples was determined after they were filtered and examined. The concentration time decay curves were produced by plotting the Ce/Co vs. time.

### Adsorption kinetics models

#### Pseudo-first-order kinetic model

This model may be expressed with reliability using the Lagergren equation. It describes how the MB adsorbed by geopolymer kinetically^45^. The Eq. (3) represents the model^46^:

3 $${\text{dq}}/{\text{dt }} = {\text{ k1}}\left( {{\text{qe }}{-}{\text{ qt}}} \right)$$

where q_t_ (mg/g) and q_e_ (mg/g) are the solute amounts adsorbed at any given time t (min) and equilibrium, respectively. The pseudo-first order rate constant is k_1_ (min^-1^). Take integration of Eq. (3), depending on the boundary conditions of q_t_ = 0 at t = 0 and q_t_ = q_t_ at t = t, the equation becomes Eq. (4) ref.^46^:

4 $${\text{ln}}({\text{q}}_{{\text{e}}} - {\text{q}}_{{\text{t}}} ) \, = {\text{ ln q}}_{{\text{e}}} - \left( {{\text{K}}_{{1}} {\text{t}}} \right)$$

The plotting of ln (qe – qt) versus yield the slope (k_1_) and intercept (ln q_e_). The equilibrium MB solute uptake (qe) and first order kinetic constant (k_1_) can eventually be determined.

#### Pseudo-second-order kinetic model

The pseudo second-order model is represented by the equation below. As illustrated below in Eq. (5), the mass transfer of driving force, (qe—qt), is proportional to the number of unoccupied sites.^47^:

5 $${\text{dq}}/{\text{dt }} = {\text{ k}}_{{2}} \left( {{\text{q}}_{{\text{e}}} {-}{\text{ q}}_{{\text{t}}} } \right)^{{2}}$$

Taking integration of Eq. (5) whilst considering boundary conditions of *qt* = 0 at *t* = 0 and *qt* = *qt* at *t* = *t*, the equation converts to Eq. (6):

6 $${1}/\left( {{\text{q}}_{{\text{e}}} {-}{\text{ q}}_{{\text{t}}} } \right) \, = { 1}/{\text{q}}_{{\text{e}}} + {\text{ k}}_{{2}} {\text{t}}$$

where k_2_ is a second-order rate constant. To illustrate a linear relationship, rearrange Eq. (6) as follows in Eq. 7:

7 $${\text{t}}/{\text{q}}_{{\text{t}}} = \, \left( {{1}/{\text{k}}_{{2}} {\text{q}}_{{\text{e}}}^{{2}} + {\text{ t}}/{\text{q}}_{{\text{e}}} } \right)$$

The connection between t/qt and t yields the slope (1/q_e_) and intercept 1/(k_2_q_e_^2^). Finally, the second order rate constant (k_2_) and the amount.

## Results and discussion

### Partially de-aluminate metakaolin (PDK) and Metakaolin

XRF analysis revealed a high fineness for PDK with an average diameter of ~ 9.5 µm, and the average diameter of MK was ~ 9.5 µm with an average diameter of ~ 4.5 µm.]. The chemical composition of metakaolin (MK) and partially de-aluminated metakaolin is shown in Table 2. The major constituents of PDK and MK were silica, and alumina. Therefore, they are a good source for the basic ingredients for geopolymer.

**Table 2:** The chemical composition of PDK, and MK.

Parameter	Amount (weight %)	
	PDK	MK
SiO_2_ (quartz)	21.2	16.3
SiO_2_ (amorphous)	63.3	41.7
Al_2_O_3_	6.2	35
Fe_2_O_3_	0.6	1.2
TiO_2_	2.8	2.6
MgO	0.11	0.15
CaO	0.13	0.51
Na_2_O	0.07	0.13
K_2_O	0.05	0.07
SO_3_	1.2	0.55
Cl^-^	0.06	0.11
LOI	3.56	1.0
Total	99.28	99.32

### SEM of PDK and MK

**Fig. 4:**
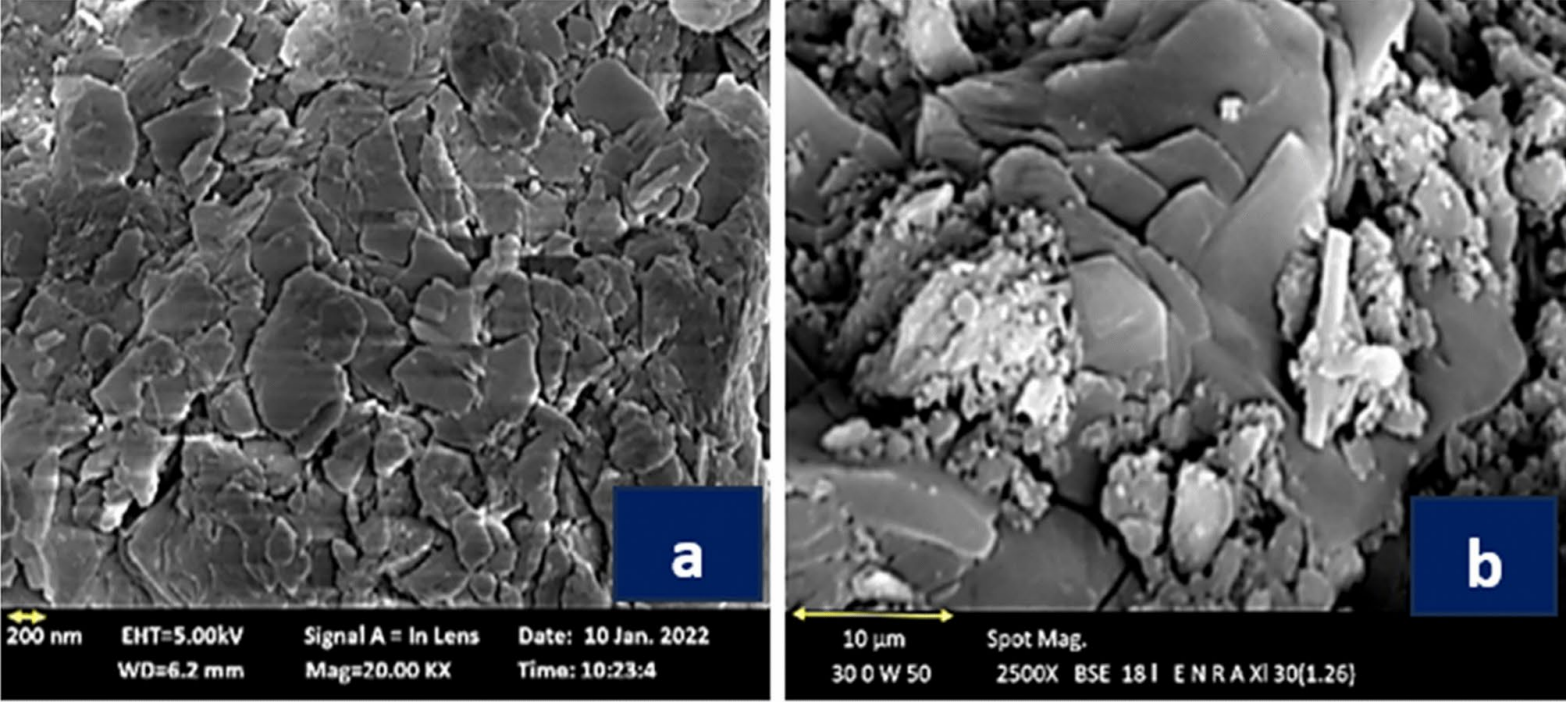
SEM of MK (**a**) and PDK (**b**).

SEM investigation was performed on MK and PDK (Fig. 4a, b). Figure 4a shows that after calcination of kaolin, the obvious layered structure of kaolin had been destroyed and replaced by the disordered scale-like arrangement structure. The layered morphology of PDK is clear, and evidences increasing of the surface area that may reflect the reactivity of PDK.

### The chemical composition of geopolymer

The prepared geopolymer that achieved higher compressive strength was as listed in Table 3. According to XRF investigation SiO_2_, Al_2_O_3_, and Na_2_O as showed in table are the primary components that are responsible for the formation of geopolymer^48^.

**Table 3:** XRF of the highest compressive strength prepared geopolymer.

The parameters (%)									
SiO_2_	Al_2_O_3_	Fe_2_O_3_	TiO_2_	Na_2_O	CaO	P_2_O_5_	Cl	Loss of Ignition	Total
55.7	20.7	1.44	2.14	7.6	0.32	0.09	0.03	16.3	99.33

### X-Ray diffraction

The amorphous phase presence suggests that the geopolymer matrix has been effectively formed, as this chaotic structure is typically associated with geopolymers. The quartz that is observed is likely a byproduct of the metakaolin or other additives, and it does not endure a complete reaction during the geopolymerization process. In these systems, quartz frequently demonstrates inertness^49,50^. The anatase phase’s existence may be attributed to the incorporation of TiO₂ in the primary materials or as an addition. In the geopolymer, the X-ray diffraction (XRD) pattern indicates the presence of unreacted crystalline phases, including anatase and quartz, as well as an amorphous geopolymer matrix (characterized by a large ridge). The material possesses a predominantly amorphous structure, which is typically found in geopolymers. Nevertheless, the geopolymer’s unreacted substances or additives are illuminated by the presence of crystalline components. as indicated in Fig. 4 ref.^51,52^.

### FTIR spectra

**Fig. 5:**
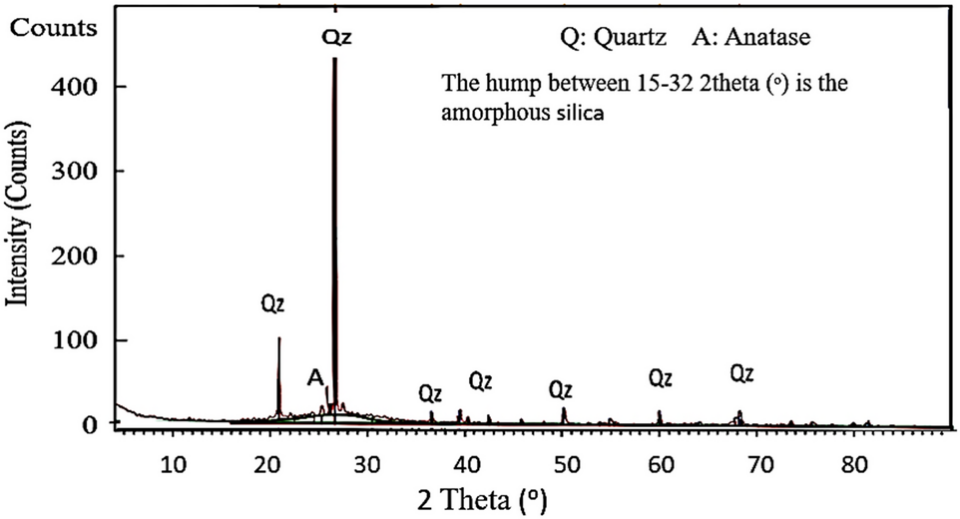
XRD of geopolymer of highest compressive strength.

The spectral pattern of the geopolymer (Fig. 5) exhibits a broad band between 3000 and 3600 cm^-1^, and another weak one located at 1647.6 cm^-1^, which are attributed respectively to the low-energy O–H bond and the H–O–H functional group, resulting from free water molecules, either sorbed on the surface or caught between the recesses of the geopolymeric framework^53,54^. The sharp absorption band appearing at 1430 cm^-1^ in the geopolymer product is related to the vibrational stretching of the O–C–O bond, due to the presence of carbonate^55^. The appearance of later band refers to the unreacted NaOH, which was combined with carbon dioxide from the surrounding medium^56^. Then, the band at 976.6 cm^-1^ corresponding to stretching vibration of Si–O-T (T = Si or Al). This change can be interpreted by the occurrence of a geopolymerization reaction that included the dissolution process and the condensation reaction forming new bonds, which form oligomers –[Si–O–Al–O–Na]- which polymerized, forming the sodium aluminum silicate hydrate gel [N-A-S–H gel]^57^.

It was also noted that the band positioned at 685.8 cm^-1^ assigned to stretching vibrations of the Si–O quartz^58,59^. These findings are in agreement with the XRD results observed above.

**Fig. 6:**
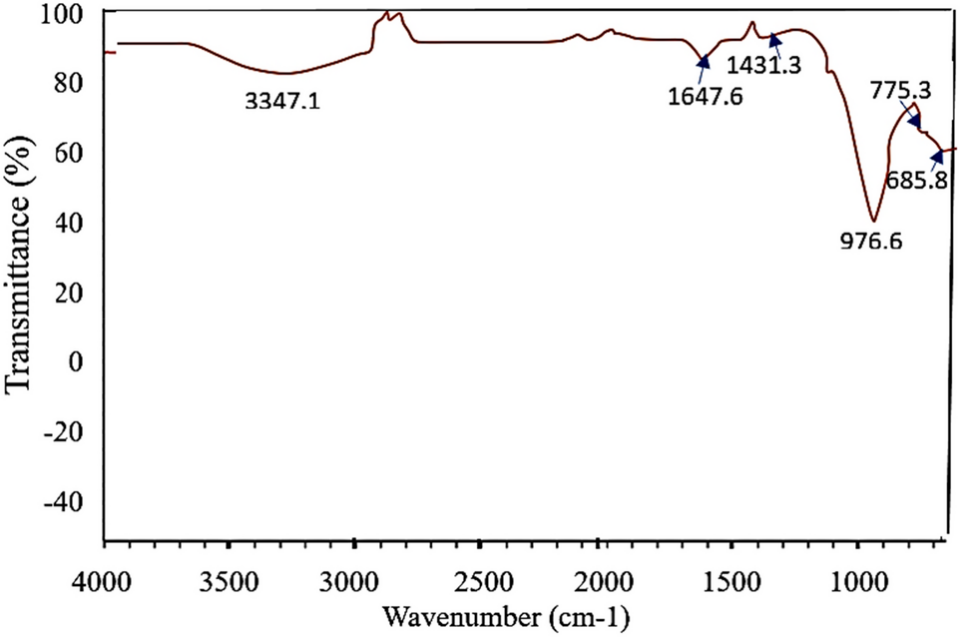
FTIR spectrum of the prepared geopolymer.

Figure 6 illustrates the Image of surface morphology of a geopolymer particle at 500 × magnification. The surface is rough and granular, like metakaolin-based geopolymers. For adsorption, roughness improves surface area for pollutant interaction like methylene blue. The selected location appears to have irregularly shaped particles or clusters. Geopolymer networks, generated by polycondensation of aluminosilicate materials (metakaolin) in an alkaline media, have this irregularity. Small particles or agglomerates on the surface indicate a heterogeneous geopolymerization structure. These tiny particles may be unreacted metakaolin or other geopolymerization phases^60^. EDX microanalysis analysed the elemental composition of metakaolin and geopolymer. EDX analysis showed that oxygen, sodium, aluminium, and silicon made up 49, 10.6, 11, and 26% respectively of the mixture. The composition of the geopolymer complies with XRF^61^.

the EDS mapping showed that the distribution of the elements in the prepared geopolymer was significantly different from the Si content due to the increased loading amount of PDK during the geopolymerization.

### Batch adsorption testing

The pH is an important parameter in determining the effect of the aqueous medium on the adsorption capacity of a material. It was known that high concentrations of H^+^ ions were at low pH (below 7). Hence, these H^+^ ions compete with MB cations on the binding sites of adsorbing material. As a result, electrostatic repulsion takes place, reducing the adsorption capacity of MB on the surface of the geopolymer. On the other size, at higher pH, a negatively charged surface of the geopolymer is formed from the predominance of OH^−^ ions in the solution. Therefore, an increase in the adsorption capacity of the geopolymers for MB ions is achieved, based on the electrostatic attraction. In this work, pH 8 was selected because this value is close to neutral, which does not need pH adjustment in the applications.

### Effect of initial concentration of MB on its removal

**Fig. 7:**
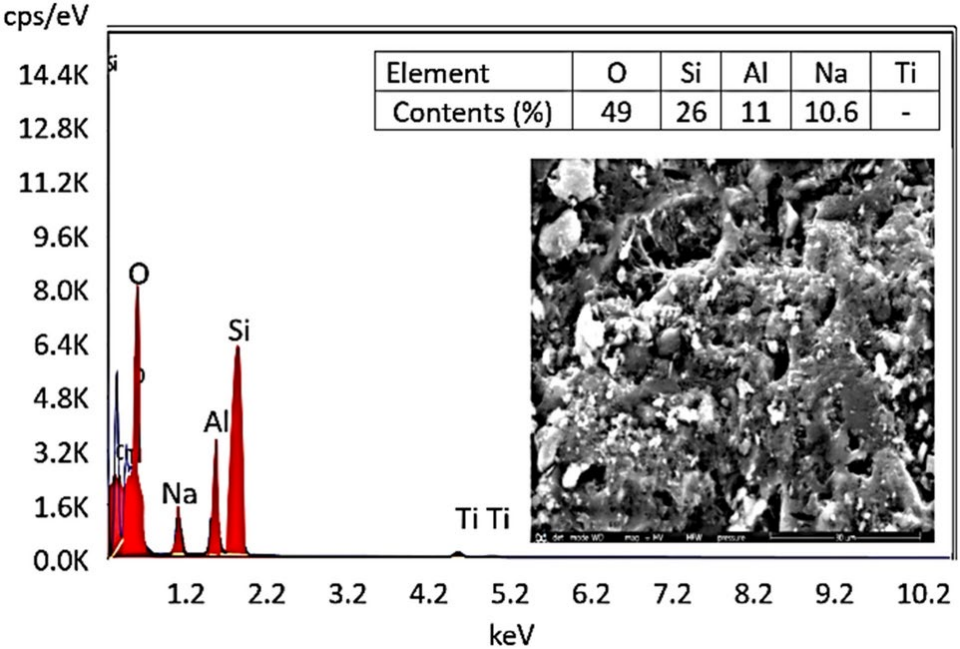
SEM − EDS mapping of the prepared geopolymer.

**Fig. 8:**
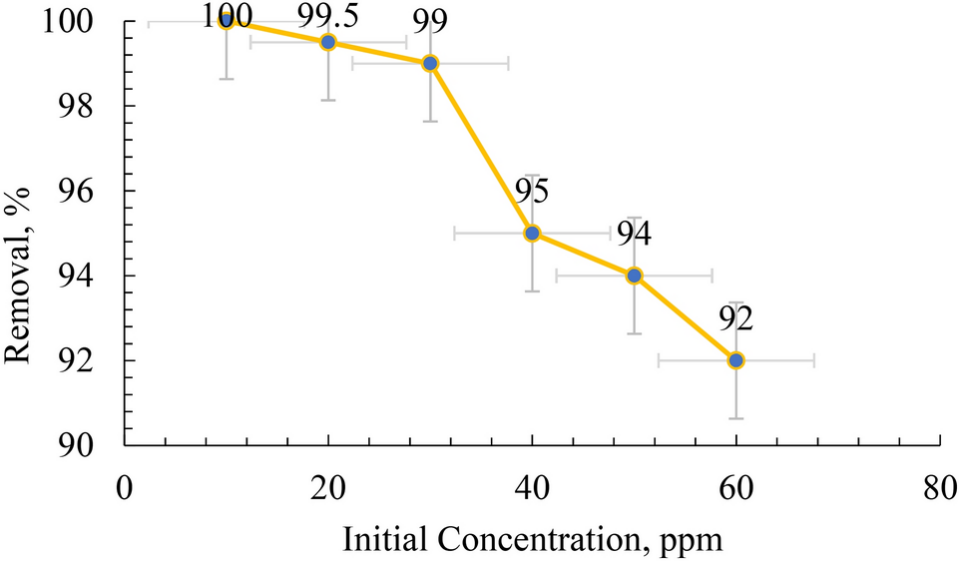
Removal percentage at different initial concentrations of MB.

**Fig. 9:**
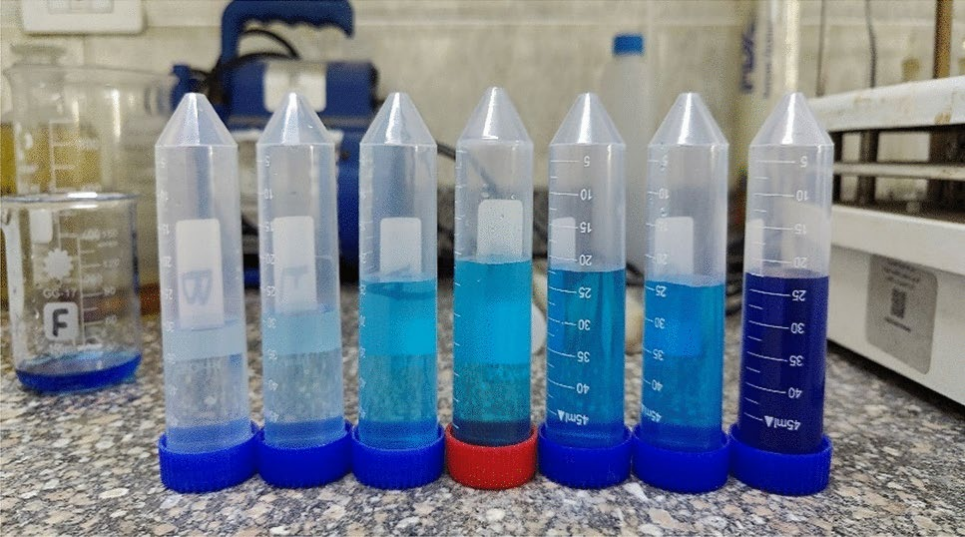
Removal of MB with different concentration by the prepared geopolymer.

The effect of initial concentration of MB on its removal by the geopolymer powders is shown in Table 4, and Figs. 7, 8 and 9. The graph shows the impact of the starting concentration of methylene blue on the effectiveness of its removal by a geopolymer of metakaolin-based composition. Once the initial concentration of MB rises from 10 to 60 ppm, there is a progressive decline in the effectiveness of removal. More precisely, the percentage of elimination decreases from just over 100% at lower concentrations (10 ppm) to 92% at the maximum dosage (60 ppm).

**Table 4:** Value of the Removal Capacity (%) and Uptake Amount (mg/g) of MB Adsorption of the Obtained Geopolymer at pH 8.

Initial concentration C_o_ (mg/L)	Removal capacity (%)	Uptake amount Q_e_ (mg/g)
10	100	1.42
20	99.5	2.8
30	99	4.2
40	95	5.4
50	94	6.7
60	92	8

This phenomenon is frequently observed in adsorption processes, where the starting concentration of the pollutant greatly influences the adsorption capacity of the adsorbent, namely the geopolymer in this instance. At reduced concentrations of MB, the abundance of adsorption sites on the geopolymer surface is adequate to capture almost all the dye molecules, leading to almost total elimination. Table 4 shows that the adsorption capacity increases as the initial concentration increases.

### Effect of geopolymer dose on MB removal

**Fig. 10:**
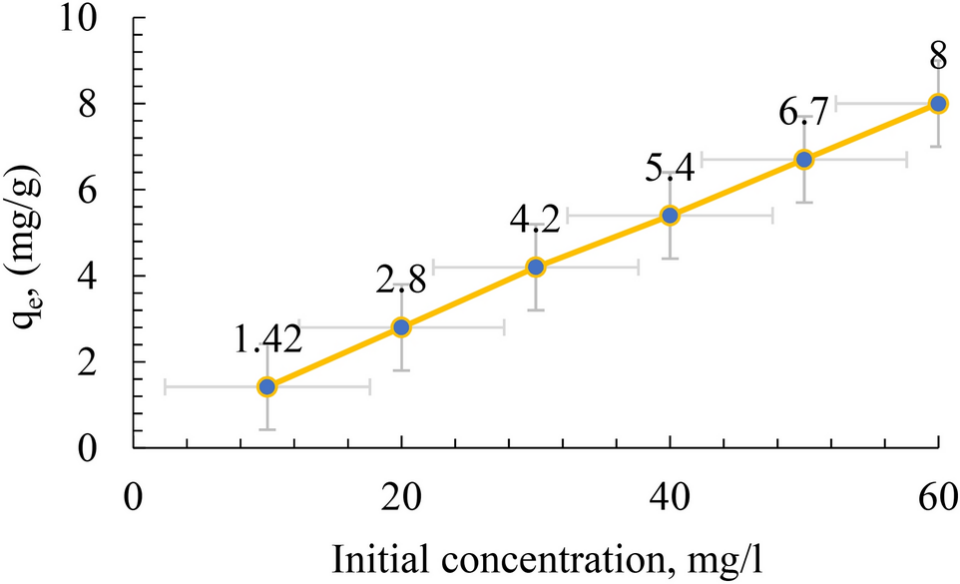
Effect of initial MB dye concentration on the adsorption capacity.

The removal of MB was examined in relation to the adsorbent dose, and the results are illustrated in Fig. 10. It is evident that when the geopolymer dose increase from 0.1 to 1 g/L, the elimination of MB increased from 70 to 88% at pH 7. These results may be attributed to the growing number of bindings sites in the geopolymer, which increases the number of adsorbents and enhances the removal of MB. Controlling the adsorption capacity greatly depends on optimizing the capacity of the adsorbent. Increasing the quantity of adsorbents may result in more binding sites in the geopolymer for MB removal. Optimizing adsorbent mass is crucial for controlling adsorption capacity.

**Fig. 11:**
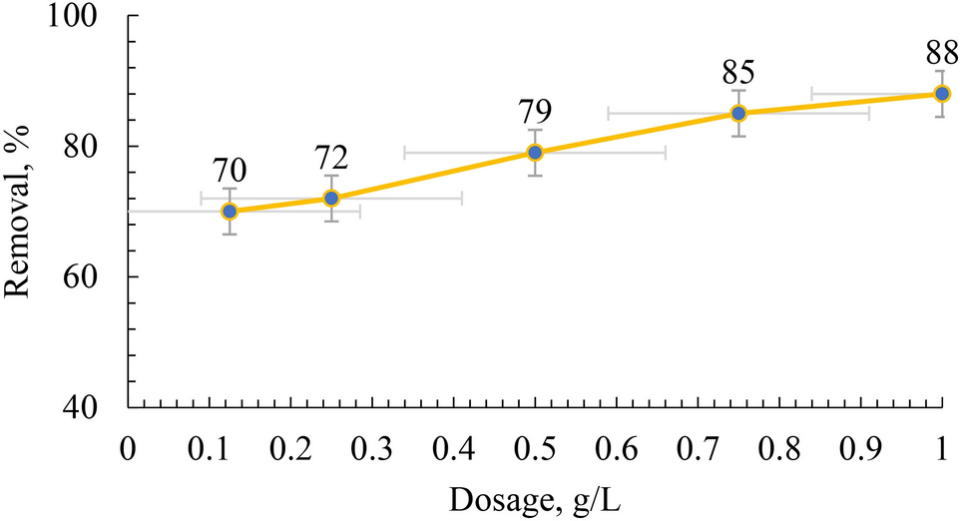
Effect of GP dose on the removal of MB at pH 7 for 60 min.

Figure 11 presents the adsorption capacity of MB as a function of adsorbent dosage at a fixed MB concentration (60 mg L^−1^) and volume (50 mL). The results reveal that the adsorption capacity obtained for 0.125 to 1.0 g dosage ranged from 6.36 to 8.0 mg. g^−1^. The overall, an upsurge in the removal and adsorption capacity is attained with the increase in adsorbent dosage, and the trend is explained as with more adsorbent, there is an increase in adsorption sites due to the presence of more adsorbent materials. The adsorption capacity increases with the higher adsorbent doses due to increased surface area, active sites availability, and interaction possibilities. This enhances the efficiency and leads to multilayer adsorption^62^.

### Effect of pH on removal

**Fig. 12:**
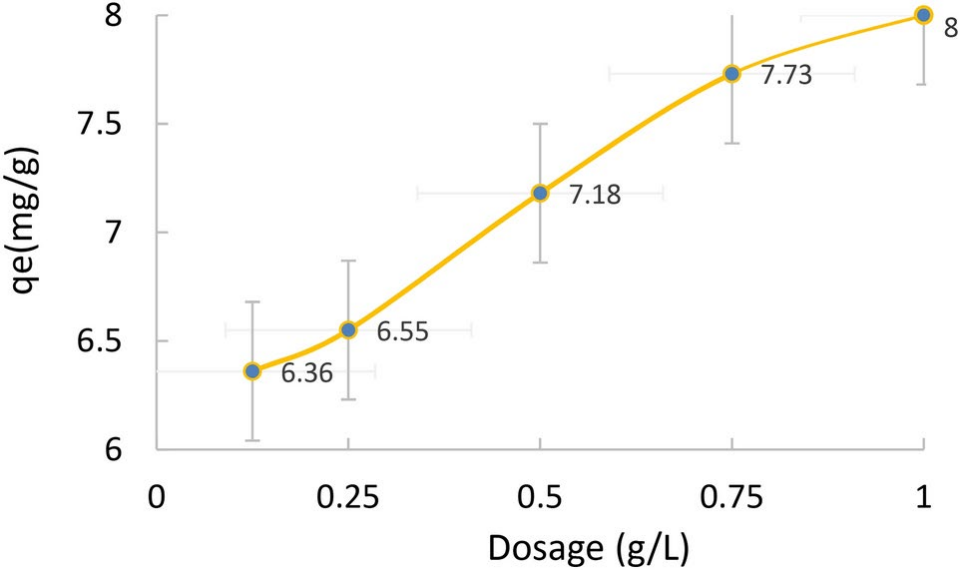
Effect of GP dose on the adsorption capacity of MB at pH 7 for 60 min.

The pH level significantly affects the elimination of organic materials from water. Figure 12 illustrates the impact of the initial pH of the solution on the adsorption process. It has been shown that the effectiveness of removal rises as the pH value increases, reaching 98.4% at a pH value of 12. The elimination process is influenced by the alteration in the pH level of the solution. When the geopolymer is in an acidic environment, the surface of the material is enveloped by H^+^ ions. This leads to a reduction in the interaction between the solute ions (MB^+^) and the sites on the geopolymeric material. In contrast, in the neutral solution, the concentration of H^+^ ions reduce, leading to a favorable interaction between the dye ions and the surface sites. The increase in removal can be attributed to the electrostatic interaction between the negatively charged particles of the geopolymer and the positively charged cationic dye. The findings indicate that the interaction between the inorganic framework (with a negative charge) and methylene blue (with a positive charge) is pH-dependent.

### Effect of contact time

**Fig. 13:**
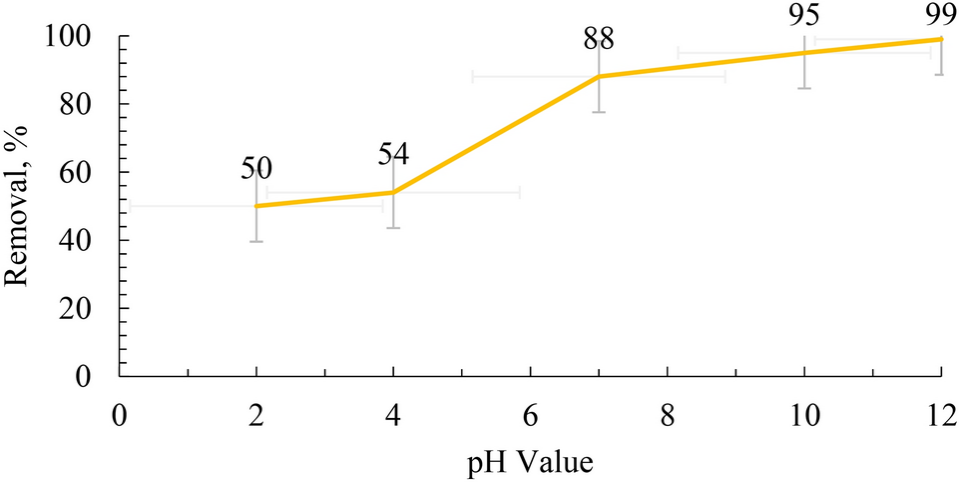
Effects of pH on the MB adsorbing by GP initial Methylene Blue (MB) concentration = 60 ppm, GP 0.35 g and temperature = 25 °C for 60 min.

The impact of contact time on the removal of MB by the geopolymer is illustrated in Fig. 13. The results indicate that the removal percentage of MB is initially rapid within the first 60 min of contact time. Subsequently, equilibrium is achieved within 180 min. After reaching equilibrium, there is no significant change in the removal percentage of the geopolymer across different dye concentrations.

The results indicated that the prepared geopolymer from PDK has achieve good performance and it can be compared with other adsorbents for removal of MB as illustrated in Table 5.

**Table 5:** The adsorption capacity of MB dye and the influence of geopolymer developed from different industrial wastes previous works and in this study.

Adsorbent Type	MB conc	Adsorbent dose	pH	Contact time	Adsorption capacity	References
-Fly ash geopolymer monoliths	1 – 50 mg/L	0.11–0.55 g	2—12	30 h	15.4 mg/g	^63^
-Fly Ash –based geopolymer spheres	(10 – 250) mg/L	(1–2.5) g/ 0.2 L	–	24 h	30.1 mg/g	^64^
-Clay and rice husk geopolymer	25 mg/L	0.1 g	3	20–80 min	15.95, 14.9, and 20.2 mg/g	^65^
-Red mud and rice husk based geopolymer composite8	(20- 120) mg/L	0.1 g	8	180 min	6.59 – 10.74 mg/g	^66^
-Partially dealuminated metakaolin and Metakaolin	(10–60) mg	0.35	7	60 min	8 mg/g	This study

### Adsorption isotherm

For studying the adsorption process, it is an important issue to investigate the equilibrium of adsorption which is normally named adsorption isotherm. The adsorption of MB dye can be upgraded by determination the equilibrium curve correlation. The isotherms of adsorption describe the equilibrium state between MB dye that is adsorbed by the sites of the GP and the MB dye amount in solution. There are number of isotherm models that describe the adsorption were adopted such as Freundlich (1907), Langmuir (1918), Redlich and Peterson (1959), Temkin, Dubinin Radushkevich. Freundlich, Langmuir are the most applied models for describing how the adsorption takes place^67^.

In this work, the adsorption results were assessed with the common models; Langmuir, Freundlich, and Temkin, to clarify the relationship between the adsorbent geopolymer and the MB dye in the solution.

**Fig. 14:**
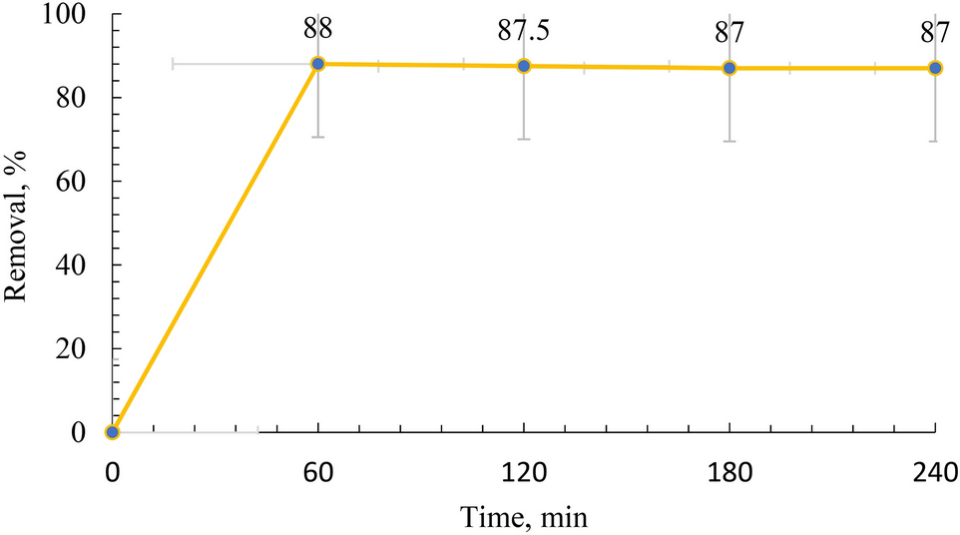
Effects of contact time on the Mb adsorbing by GP (pH = 7, initial Methylene Blue (MB) concentration = 60 ppm, GP 0.35 g and temperature = 25 °C.

**Langmuir isothermal model** describe of the monolayer adsorption and the homogeneity of surface sites^42^. For the adsorption occurring without reactions between the adsorbent species, the straight-line relationship of Langmuir was calculated by the following Eq. (8):

8 $${\text{C}}_{\text{e}}/{\text{q}}_{\text{e}}=\text{q}/{\text{K}}_{\text{L}} {\text{q}}_{\text{m}}+{\text{C}}_{\text{e}}/{\text{q}}_{\text{m}}$$

whereas C_e_ is the concentration of MB dye in the solution at the equilibrium (mg/L), q_e_ is the capacity of adsorption per unit mass at the equilibrium (mg/g), q_m_ is the maximum adsorbate per unit mass of adsorbent after complete coverage of one layer on geopolymer (mg/g), and K_L_ is the Langmuir constant (L/mg). Plotting C_e_/q_e_ versus C_e_ by using geopolymer is shown in Fig. 14a. The obtained data shows that the linear regression coefficient R^2^ value = 0. 886 for the model of Langmuir^23,68–70^. The dimensionless constant or the equilibrium parameters (R_L_) is one of the most important parameters of the Langmuir model, which reflects the nature of the adsorption process. R_L_ value is calculated from Eq. (9) ref.^37,38^.

9 $${\text{R}}_{{\text{L}}} = 1/(1 + {\text{R}}_{{\text{L}}} {\text{C}}_{{\text{o}}} )$$

Where R_L_ is the Langmuir constant and C_0_ is the initial concentration of MB dye. The calculated R_L_ value of MB dye is between zero and one (0 < R_L_ < 1). Such a result illustrates that the absorbability of MB is favorable by using geopolymer.

The isotherms of MB dye adsorption on geopolymer are illustrated in Figs. 14a, b and c, and the regression coefficient are depicted in the related figures.

**Freundlich isothermal model** suppose that the sites of adsorption are heterogeneous and the adsorbing materials take up the strong available binding sites quickly. The linear regression relationship can be represented by the logarithmic Eq. (10):

10 $${\text{logq}}_{\text{e}}=\left(1/\text{n}\right){\text{logC}}_{\text{e}}+{\text{logK}}_{\text{F}}$$

whereas K_F_ is Freundlich model constant which expresses the capacity of adsorption and n is the heterogeneity factor that predicts the occurrence of adsorption, whereas if n is greater than1, the adsorption proceeds properly and effectively. The values of K_F_ and n can be determined from the linear relationship plot of log q_e_ versus log C_e_, taking the intercept and the slope of the line. The 1/n value obtained from the Freundlich isotherm plot is 0.68 which falls between 0 and 1 indicating the adsorption is linear and uniform throughout the surface. According to the 1/n value (0˂1/n ˃1), this isotherm is a favourable physical process Fig. 14b).

Figures 14a, b, and c display the correlation factor (R^2^) conforming to the models Freundlich, Langmuir, and Temkin. The results confirm that Freundlich isotherm model was evaluated to be better, which has a higher coefficient R^2^ value (0.996), while the Langmuir model was 0.886, and Temkin was 0.93. Comparing these data with that of Langmuir, it can be concluded that the Langmuir model has the lowest probability, whereas the R^2^ value is the lowest of all models.

**Temkin isotherm** considers the reactions between adsorbate and adsorbent. The linear equation can be calculated from Eq. (11).

11 $${\text{q}}_{\text{e}}={\text{R}}_{\text{T}/}{\text{b}}_{\text{T}}.{\text{lnC}}_{\text{e}}+{\text{R}}_{\text{T}}/{\text{b}}_{\text{T}}{\text{lnK}}_{\text{T}}$$

whereas K_T_ is the Temkin isotherm constant (L.g^-1^), b_T_ is a constant of heat of adsorption, R is the constant of universal gas (8.314 J.mol^-1^. K^-1^) and T is the temperature in Kelvin (K), The calculated maximum adsorption capacity (q_max_, Cal) by using geopolymer is 8 mg/g for the adsorption rate of MB dye.

Table 6 summarizes all the parameters and their respective determination coefficients. Based on the values of the coefficient R^2^ (0.996) of the linear regression, the equilibrium data fit the model of Freundlich, rather than Langmuir or Temkin. For forecasting the adsorbability occurring in multilayers and for determining the adsorption capacity, the Freundlich model (Fig. 14b) was used herein^24,25^. The value of n greater than unity indicates a favourable adsorption taking place, this indicated that the adsorption occurs under a multilayer and heterogeneous surface for MB.

**Table 6:** Isotherm models parameters for Methylene blue.

Model	Parameter	MB
Langmuir	qm(mg/g)	8.0
	K_L_ (L/mg)	0.1
	R^2^	0.886
Freundlich	n	1.47
	R^2^	0.996
Temkin	R^2^	0.93
	K_T_	1.43

### Adsorption kinetics

The adsorption kinetics was assessed by conducting several experiments with different time periods. 0.35 mg of geopolymer was contacted with 50 mL of MB dye solution containing 60 mg/L of the dye, the mixture was stirred for different contact periods from 20 to 60 min at the ambient temperature. Figure 13 displayed that the adsorption of the MB dye by geopolymer proceeds fast through the first 60 min of the trial and continued slowly through the period from 60 to 240 min until the adsorption attained an equilibrium state within 240 min. These results are in agreement with those obtained by several researchers^39,40^. In the beginning the MB dye species adsorbed on the available sites for adsorption of the GP. This is rapid adsorption step due to the most active sites of the GP are free of MB. After occupation of the most active sites, the attraction force between the MB molecules with the GP gradually lowered and the rate of adsorption decreased. After filling the outer sites of the GP, the process goes through the GP taking a relatively longer time^42^.

**Fig. 15:**
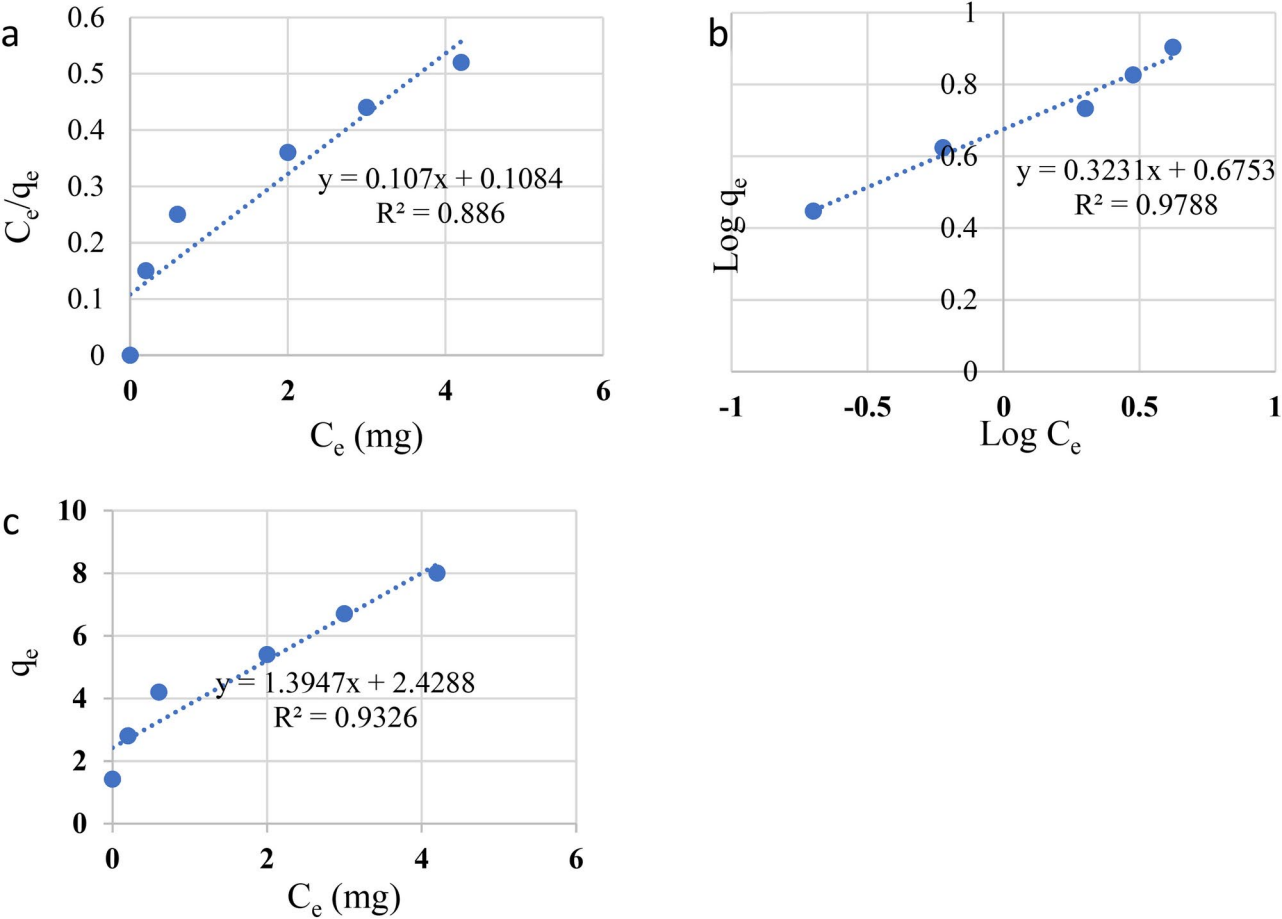
The linear forms of adsorption isotherms fitted the equilibrium states: (**a**) for Langmuir (**b**) for Freundlich and (**c**) for Temkin.

The adsorption kinetics of MB onto GP was investigated using pseudo first order, pseudo second order models as shown in (Fig. 15a and b respectively, using the experimental data at various initial concentrations. The calculated parameters values obtained from the application of the two models are indicated in Table 7. By comparing R^2^ for each applied model and the compatibility between the estimated and observed qe values, the most favourable model may be identified. The pseudo 2nd order R^2^ (09,795) value are greater than other model (0.7812), and the qe cal values for MB are considerably closer to the qe experiment for MB than other kinetic models.

**Table 7:** Kinetic models parameters for Methylene blue.

Model	Parameter	MB
Pseudo-first order	K_t_ (1/min)	− 0.215
	R^2^	0.7812
Pseudo-second order	q_e_ (mg/g)	9.345
	R^2^	0.9795

**Fig. 16:**
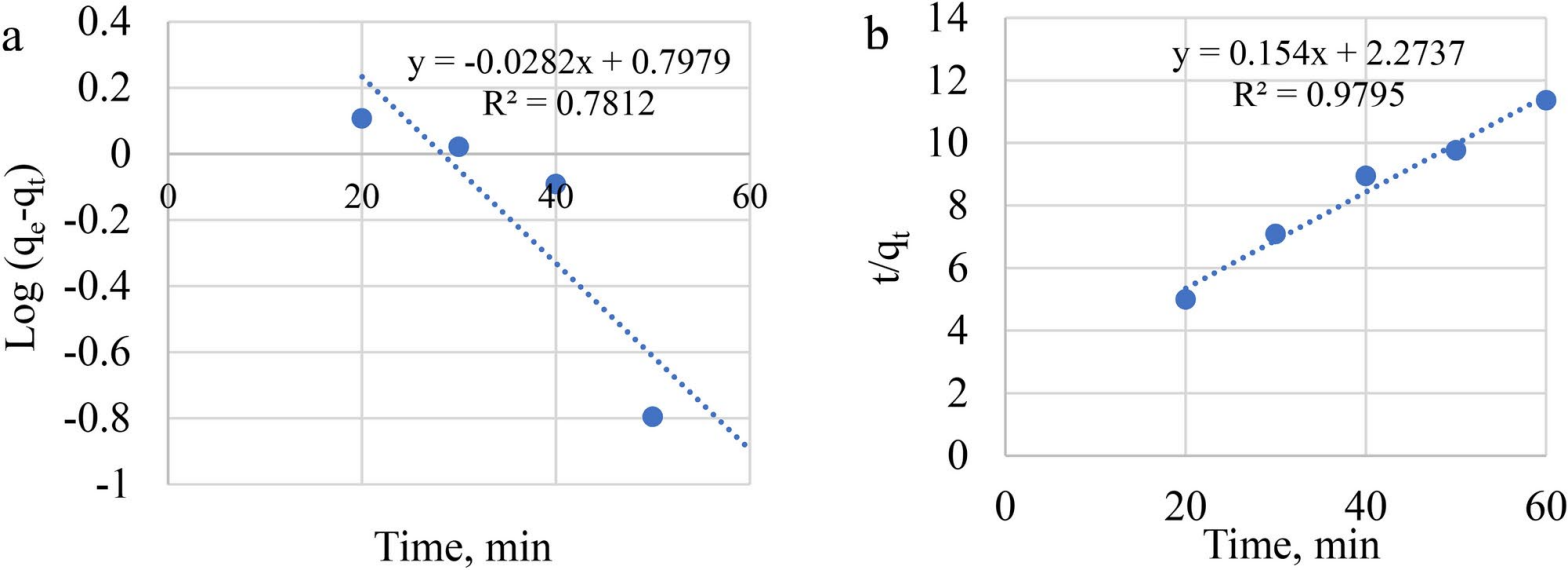
The relation between adsorption capacity and contact time. (**a**) for pseudo first order, and (**b**) for pseudo second order.

The connection between t/qt and t (Fig. 15 b) yields the slope (1/q_e_) and intercept 1/(k_2_q_e_^2^). Finally, the second order rate constant (k_2_) and the amount of adsorbed dye per mass of composite at the final equilibrium point (q_e_) were determined (Fig. 16).

## Conclusions

This study successfully synthesized a geopolymer from partially dealuminated metakaolin (PDK) as a novel solid adsorbent for the effective removal of methylene blue (MB) from wastewater. The geopolymer was prepared using NaOH1(12M) solution and PDK as alkaline activator. The geopolymer specific surface area was 9.3 m^2^ /g, which offered enough sites for the mass transfer and adsorption processes. At the optimal conditions of 25 ℃ temperature, 60 mg/L initial concentration, at pH 7, and 240-min contact time, the geopolymer adsorbent particles demonstrated high adsorption efficiency for MB dye removal, with a maximum adsorption capacity reaching 8 mg/g with a removal efficiency of 99%. The Kinetic studies revealed that the adsorption process follows the pseudo-second-order model, indicating a chemisorption mechanism. The equilibrium data fit the Freundlich isotherm model, suggesting the adsorption occurs on a heterogeneous surface with a multilayer process. The research highlights the potential of PDK-based geopolymer as a cost-effective and environmentally friendly adsorbent for wastewater treatment, offering a sustainable approach to managing industrial by-products and addressing water pollution.

## Data Availability

All data generated or analysed during this study are included in this published article.
